# Plant food allergy in mugwort sensitised patients: two case reports

**DOI:** 10.1186/2045-7022-1-S1-P81

**Published:** 2011-08-12

**Authors:** Eugenia Almeida, Isabel Carrapatoso, Filipa Ribeiro, Nuno Sousa, Antonio Segorbe Luís

**Affiliations:** 1 Coimbra University Hospitals, Immunoallergology Department, Coimbra, Portugal

## Background

Allergy to celery, other vegetables and spices from Apiaceae family is frequently seen in pollen allergic patients and can be associated with several immediate symptoms, from oral allergy syndrome to anaphylactic reactions. The association with spices belonging to a different family is frequently seen in celery allergic patients included in celery-mugwort-spice syndrome.

## Case reports

The authors describe two clinical cases.

### Case 1

Thirty one years old male that presented at age 15 allergic rhinoconjunctivitis and cutaneous pruritus after ingestion of honey and spices. Three years later, he had the same symptoms with ingestion of pepper. Skin prick tests were positive to grass, mugwort, dandelion and ragweed pollen, as well as pepper, curry, paprika and anise. Serum specific IgE was positive to carrot, pea, white bean and chick pea, mugwort, dandelion and grass. ImmunoCAP ISACR was positive to Art v1, Art v 3, Phl p 1 and Cyn d 1.

### Case 2

Twenty two years old male with history of anaphylactic reactions (urticaria, angioedema and loss of consciousness) after the ingestion of spicy food and pizza. The systemic episodes developed only after physical activity (30 to 60 minutes). SPT were positive to grass, mugwort, olive and plantain pollen, as well as tomato, capsicum, curry and nuts. Prick-prick tests were positive to celery, carrot and peach. Specific IgE was positive to mugwort, plantain, timothy, rye, olive, peach, celery, nut, hazelnut, peanut, garlic and onion. ImmunoCAP ISACR was positive to Phl p1, Phl p 11, Pru p3, Art v 3 and Cor a 8.

## Conclusions

In case 1 allergy to spices appears to be a consequence of pollen sensitisation to mugwort, plantain and dandelion with involvement of Art v1 and the LTP Art v 3. In the case 2 hypersensitivity to mugwort is related to sensitisation to LTP (Art v 3) which seems to cross-react with LTP from peach and hazelnut, causing an anaphylactic reaction. Two different patterns of sensitisation to mugwort are observed in these patients.

